# Identification and determination of ergot alkaloids in Morning Glory cultivars

**DOI:** 10.1007/s00216-016-9322-5

**Published:** 2016-02-12

**Authors:** Julia Nowak, Michał Woźniakiewicz, Piotr Klepacki, Anna Sowa, Paweł Kościelniak

**Affiliations:** Laboratory for Forensic Chemistry, Department of Analytical Chemistry, Faculty of Chemistry, Jagiellonian University in Kraków, Ingardena 3, 30-060 Kraków, Poland; Institute of Botany, Faculty of Biology and Earth Sciences, Jagiellonian University in Kraków, Kopernika 27, 31-501 Krakow, Poland

**Keywords:** Ergine, Ergometrine, Morning Glory, *Ipomoea*, UAE, LC-MS

## Abstract

Seeds of plants from *Ipomoea* genera contain numerous ergot alkaloids, including psychoactive ergine and ergometrine, and are often abused as so-called “legal highs.” In this work, an analytical method for determination of ergine and ergometrine, and identification of other alkaloids was developed, optimized, and validated. Three extraction techniques, ultrasound-assisted extraction in bath, or with sonotrode, and microwave-assisted extraction were evaluated, and it was concluded that ultrasonic bath is the most suitable technique for extraction of ergot alkaloids. The extraction method was later optimized using a Doehlert experimental design with response surface methodology and used together with the optimized LC-Q-TOF-MS method. The analytical procedure was validated in terms of recovery and matrix effect, repeatability, and intermediate precision. Limits of detection and quantification were 1.0 and 3.0 ng mL^–1^, respectively, and were sufficient for determination of ergot alkaloids in *Ipomoea* seeds. The analysis revealed that from five kinds of seeds purchased from different vendors, only three contained ergot alkaloids. Concentration of alkaloids and their relative abundance was similar in samples representative for whole seeds packs; however, when single seeds were analyzed, significant discrepancies in ergine and ergometrine concentrations were detected.

**Electronic supplementary material:**

The online version of this article (doi:10.1007/s00216-016-9322-5) contains supplementary material, which is available to authorized users.

**Graphical Abstract:**
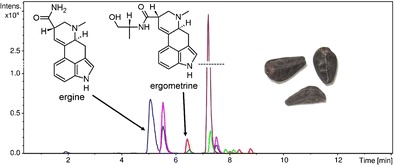
Identification of six ergot alkaloids and determination of ergine and ergometrine in Morning glory seeds using the ultrasound-assisted extraction followed the LC-MS analysis

## Introduction

The relatively strict Polish drug law, which penalizes the possession of any substance listed in the Act of July 29, 2005 on counteracting drug addiction appendix [1], causes people experimenting with psychoactive substances to take special interest in so-called legal highs. Those can be both specific compounds and plants, which give similar psychoactive effects to illicit drugs. Plants containing ergot alkaloids can be an example.

**Fig. 1:**
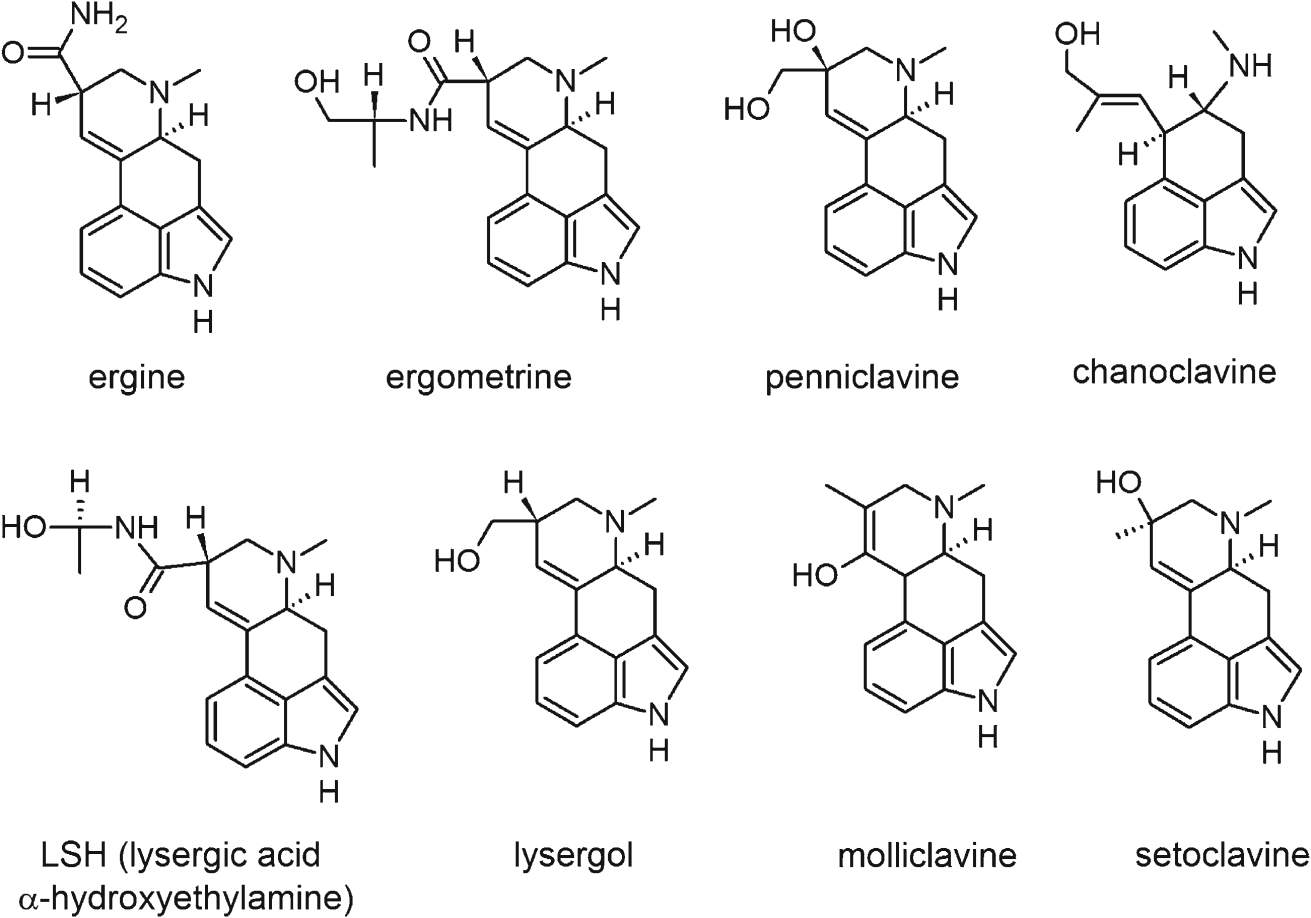
Chemical structures of some ergot alkaloids reported in *Ipomoea* seeds

Ergot (ergoline) alkaloids (Fig. 1) are present in some plants from the *Convolvulaceae* family because of their symbiosis with *Claviceps* fungi [2]. Endophytic and epibiotic fungi producing alkaloids associated with a few *Ipomea* species were described [2–4]. The most important ergoline derivative in *Convolvulaceae* is ergine (D-lysergic acid amide, LSA), together with its epimer isoergine (*iso*-LSA), which has psychoactive effect similar to D-lysergic acid diethylamide (LSD), but with more severe side-effects [5–7] and yet undiscovered mechanism of action [8]. Also ergometrine—with its diastereoisomers—is known to be biologically active [9] and other ergot alkaloids present in *Convolvulaceae* may have biological effect in humans, because of their structural similarity to LSD [8]. The highest concentration of LSA can be found in seeds of *Rivea corymbosa, Ipomoea violacea*, and *Argyreia nervosa* (Hawaiian Baby Woodrose) species [10], the latter being a popular legal high [11]. Those plants have been banned in Poland since 2009. However, ergot alkaloids are also present in lower concentration in plants from *Ipomoea* genera [6], known under the common name of Morning Glory, which are legal in Poland and in other European countries, and are widely available as ornamental plants. Therefore, Morning Glory seeds are used as a substitute for both LSD and illegal *Convolvulaceae* plants. Information on Morning Glory abuse can be found on on-line forums concerning psychoactive substances. Moreover, there are reported cases of severe intoxication and poisonings [12]. For this reason, there is a need for an effective method of identification and possibly determination of ergot alkaloids, particularly in small sample amounts, which can be left after administration of Morning Glory seeds.

Methods of determination of ergot alkaloids from other plants infected by *Claviceps* fungi, such as grass and cereals, require a large amount of sample and a laborious extraction procedure [13–16]. In recent works of Paulke et al. with detailed qualitative analysis of ergot alkaloids in *Argyreia nervosa* seeds, a simple but time-consuming maceration was used [17, 18], with no information about efficiency of the process. To the authors’ best knowledge, advanced extraction methods, such as microwave-assisted extraction (MAE) or ultrasound-assisted extraction in ultrasound bath (UAE-B) or with the use of sonotrode (UAE-S) have not yet been used for analytical purposes. UAE is a well-known extraction method and both ultrasonic bath and probe have been successfully applied for the extraction of psychoactive compounds from plant material [19–21]. MAE, which utilizes the energy of electromagnetic waves to enhance the extraction process, has also been used in such analysis and in some cases it proved to be more effective [22]. The MAE technique was successfully utilized for the preparative extraction of heat- and light-sensitive lysergol from *Ipomoea* seeds [23]; however, the decomposition of analytes induced by microwaves should always be considered at the method development stage.

In this work, we present our consideration on the extraction of ergine and ergometrine from *Ipomea* seeds, which led us to establish an effective, rapid extraction procedure using UAE in ultrasonic bath and a validated UAE-B/LC-MS method for determination of ergine and ergometrine, as well as identification of other ergot alkaloids present in less than one seed of Morning Glory. The developed method was also successfully used for the analysis of green parts of *Ipomoea* plants.

## Materials and methods

### Materials

Ergine and ergometrine maleate were purchased from THC Pharm GmbH (Frankfurt, Germany) and LGC Standards (London, UK), respectively. Methanol, acetonitrile, isopropanol (all MS purity grade), formic acid (98 % purity, MS grade), and amitriptyline (IS) were purchased from Sigma-Aldrich (St. Louis, MO, USA). Graphitized carbon was ordered from Agilent Technologies (Santa Clara, CA, USA). Ultrapure water (18.2 MΩ∙cm, 3 ppb TOC) was generated in our laboratory in a Milli-Q system by Merck-Millipore (Darmstadt, Germany).

Samples of *Ipomoea* seeds of different species and cultivars were purchased from local and on-line ornamental plant shops. They were: *Ipomoea purpurea* “Morning Call” (IP-MC), *Ipomoea purpurea* without cultivar name (IP), and two *Ipomoea tricolor* “Heavenly Blue” from different vendors (IT-HB1 and IT-HB2). Moreover, a sample of *Ipomoea purpurea* “Heavenly Blue” (IP-HB) was purchased from an on-line shop magicznyogrod.pl.

One hundred IT-HB2 seeds were sown in the tropical glasshouse at the Botanical Garden of Jagiellonian University in Kraków, Poland, in turf-based substrate for sowing seeds (pH 6–7). After 1 wk, 95 % of seeds germinated but soon the plants were attacked by seedling blight and were treated with antifungal agent (Previcur Energy 840 SL) for 2 wk. After the treatment, the seedlings were healthy without any signs of disease or physiological damage. Young, non-flowering plants (40-d old) were cut and dried at room temperature.

As deuterated standards of ergot alkaloids were not available in economically reasonable scale, amitriptyline was chosen as IS. Amitriptyline has certain similarities to ergot alkaloids in both chemical structure and properties; it is also stabile in investigated extraction conditions and it is definitely not present in plant material.

### Preparation of stock and standard solutions

Stock solutions of ergine (10 mg mL^–1^), ergometrine maleate (13.6 mg mL^–1^, an equivalent of 10 mg mL^–1^ ergometrine), and stock solution of IS (10 mg mL^–1^) were prepared in methanol and stored at –20 °C. Standard solutions were prepared in methanol:water (7:3, v/v) mixture with 50 ng mL^–1^ IS and diluted 1:1 with ultrapure water prior to the analysis to obtain the following calibrators: 0.5, 1, 5, 10.0, 20, 50, 100, 200, 300, 500 ng mL^–1^ of ergine and ergometrine; the concentration of IS was always 25 ng mL^–1^. Standard solutions were kept at +15 °C and were analyzed within less than 48 h since preparation.

### Instrumentation

An XA 220/X analytical balance equipped with a DJ-02 antistatic ionizer (Radwag, Poland) was used in order to minimize the electrostatic force influence on powdered plant tissue samples and, in this way, to minimize weighing error. MAE was carried out in a CEM 5 microwave-assisted sample preparation system (CEM; Matthews, NC, USA) equipped with Xpress PFA extraction vessels (75 mL capacity). UAE-B was performed using Sonic 3 ultrasonic Bath of 310 W (Polsonic, Poland), and UAE-S was performed using the Vibra Cell VC 50 device of 50 W equipped with a sonotrode (Sonic and Materials Inc., CT, USA).

The LC-MS analyses were carried out on an UltiMate 3000 RS liquid chromatography system (Dionex, Sunnyvale, CA, USA) coupled to a mass spectrometer with ESI ion source and quadrupole-time of flight mass analyzers (MicrOTOF-Q II; Bruker, Bremen, Germany). For optimized chromatographic separation, a C18 Ascentis Express column (100 × 2.1 mm, 2.7 μm; Supelco, Bellefonte, PA, USA) was used. The mobile phase consisted of acetonitrile and 0.1 % formic acid delivered in a gradient program (0 min ACN: 5 %, 0.5 min ACN: 5 %, 8 min ACN: 25 %, 10 min ACN: 90 %, 11.5 min ACN: 90 %, 13 min ACN: 5 % with 4 min post-run for column equilibration) at a flow rate of 0.3 mL min^–1^. The autosampler and the column were thermostated at +15 °C and +20 °C, correspondingly. Injections of 3 μL were repeated twice for standards and three times for samples analyses.

ESI+ ion source operated at +180 °C, 6 kV, with nebulizer gas pressure set up at 0.3 bar and dry gas flow set up at 7 L min^–1^. Mass analyzers were working at hexapole rf 50 V_pp_, collision rf 400 V_pp_, transfer time 50 μs, and pre-pulse storage 5 μs: in full scan MS mode (150–1000 *m/z* range), collision energy 8 eV for quantitative analysis, whereas for qualitative analysis: in untargeted Auto-MS/MS mode with collision energy of 20 eV for identification of the unknowns and multiple reaction monitoring (MRM) mode for confirmation of initially identified compounds and untargeted Auto-MS/MS mode with collision energy of 20 eV for identification of the unknowns. MRM is a specific MS/MS mode available in a Bruker Q-TOF MS system, which enables isolation of precursor ion, its fragmentation, and recording the fragment ions spectra in full scan range. Before each run, the TOF detector was calibrated using isopropanol-sodium formate clusters, according to the procedure given by the manufacturer.

### Sample preparation

Samples of seeds were ground finely in a ball mill and weighed carefully to 10.0 ± 0.5 mg. Two kinds of samples were prepared: samples of single seeds of a given type and samples representative for the whole pack of seeds. The latter were prepared by grinding 1.5–2 g of seeds randomly selected from whole seeds pack. 10 mL of any tested extracting solution with 50 ng mL^–1^ IS was added to the ground seeds. Such prepared system was ready for subsequent extraction. The extracts were then cooled to room temperature. Seed extracts were centrifuged, filtered through a 0.45 μm regenerated cellulose syringe filter, diluted 1:1 with ultrapure water, and analyzed directly. For optimization, qualitative/quantitative analysis samples were prepared in three replicates (n = 3), but four replicates (n = 4) were investigated during the validation study.

### Extraction method optimization

For optimization of extraction method, three techniques were evaluated: MAE, UAE-B, and UAE-S. In preliminary experiments conducted using the UAE-B technique, the sample and solvent amount as well as the extracting solvent type were evaluated.

After preliminary experiments for assessing the amount of sample, solvent, and the solvent type, the three-factor Doehlert experimental design with response surface methodology (RSM) was used in order to optimize three main factors for MAE and UAE-B: time, temperature, and solvent composition: methanol to water content. Doehlert experimental design enables the analysis of each factor on multiple levels, distributed evenly thorough optimization range, and thus enables empirical construction of a second-order model [24]. It is also one of the most efficient experimental designs, as for the three-factorial model, only 13 experiments with different factor values are required. In this case, a quadratic polynomial function was formulated as the model, using response surface regression, where response was calculated as the ratio of ergine/ergometrine to IS peak area. It was therefore directly connected with extraction efficiency. Then, the desirability function D was constructed as the normalized sum of ergine and ergometrine response models, so that it would have value 1 for best parameter values (highest extraction efficiency) and 0 for unfavorable ones.

For MAE, time was optimized in the range from 5 to 35 min, at 7 levels, temperature in the range from 45 to 75 °C at 5 levels, and solvent composition from 50 to 100 % of methanol at three levels. As MAE oven is equipped with airtight vessels, it is possible to exceed the methanol boiling point during extraction procedure. Coded and experimental values of parameters in Doehlert design are presented in the Electronic Supplementary Material (ESM) in Table S1.

For UAE-B, time was optimized in the range from 5 to 35 min, at seven levels, temperature in the range from 40 to 60 °C at five levels, and solvent composition from 50 to 100 % of methanol at three levels. In case of UAE-B, methanol boiling point could not be exceeded, as it could lead to depressurization of extraction vials (see Table S2 in the ESM for details).

UAE-S has to be performed in open vessels, and due to the excessive heat production during extraction, there is no feasible way to control solvent temperature. As it was assumed that optimal solvent composition should be the same as in the case of UAE-B, only time of extraction was optimized by the single variable optimization, in the range from 0.5 to 3 min and 0.5 min intervals. Temperature of the solvent was measured directly after every extraction to assure that it does not reach the boiling point of the solvent.

### Validation procedure

Validation was performed following rules of validation guideline for food analysis and bioanalysis [25–27]. The linearity of the method was tested using the set of calibrators: 0.5, 1.0, 5.0, 10, 20, 50, 100, 200, 300, 500, 1000 ng mL^–1^ of ergine and ergometrine, taking as a signal relative peak area (target/IS). The weighed (1/x^2^) linear calibration model was applied. The calibration residuals analysis (fitting ±15 % range) was chosen as the criterion of acceptance. The precision was assessed in terms of repeatability (n = 4) and intermediate precision (3 consecutive d, 4 samples each d) using the one-way ANOVA approach [28].

The recovery and matrix effect were evaluated according to the method proposed by Matuszewski et al. [29]. As ergot alkaloids are naturally occurring constituents of *Ipomoea* seeds infected by fungi and there are no blank samples, matrix effect (ME%), recovery (RE%), repeatability, and intermediate precision were calculated for the analyte concentration in the sample with the standard added on three levels: +25 %, +50 %, and +75 % of analyte concentration in the sample (an equivalent of 10, 20, 30 μg g^–1^ of ergometrine and 90, 160, 240 μg g^–1^ of ergometrine, respectively). In case of matrix effect, a standard was added to the sample after the entire preparation procedure, directly before analysis, and in case of other parameters, standard was added to the sample before extraction. ME% and RE% were calculated for every level according to Eqs. 1 and 2, respectively:

1 $$ ME\%=\frac{B}{A}\kern0.3em \cdot \kern0.3em 100\% $$

2 $$ RE\%=\frac{C}{B}\kern0.3em \cdot \kern0.3em 100\% $$

where *A* is the concentration of the added standard; *B* is the difference of concentration between extract spiked after extraction and extract without standard addition; *C* is the difference of concentration between extract spiked before extraction and extract without standard addition. Repeatability and intermediate precision were calculated using within- and between-day variations, calculated by the ANOVA approach [30], according to Eqs. 3 and 4, respectively:

3 $$ \mathrm{R}\mathrm{e} peatability=\frac{\sqrt{WMS}}{\overline{c}}\kern0.3em \cdot \kern0.3em 100\% $$

4 $$ Intermediate\kern0.5em  precision\kern0.5em =\frac{\sqrt{E(BMS)}}{\overline{c}}\kern0.5em \cdot \kern0.3em 100\% $$

where *WMS* is the within-d mean square; *E(BMS)* is the between-d variance; $\overline{c}$ is the mean concentration of a given compound. The criterion of acceptance was set up to 80–115 % for recovery and matrix effect (for individual assays) and ±15 % for repeatability and intermediate precision.

Limit of detection (LOD) and limit of quantification (LOQ) were estimated by systematic dilution of standard solutions (ng mL^–1^) till the criterion of signal to noise ratio (S/N), with respect to S/N = 3 and S/N = 10 were met, for LOD and LOQ respectively. The LOD and LOQ were then recalculated for solid samples (expressed in μg g^–1^) taking into account the sample mass (10 mg). Subsequently, after analysis of Morning Glory seeds (see section “Analysis of different *Ipomoea* seeds”), estimation of LOD and LOQ were verified by spiking the most similar matrix (i.e., IP seeds not containing ergine and ergometrine), to the following levels: 0.5, 1.0, 2.0, 3.0, 4.0, and 5.0 μg g^–1^.

## Results and discussion

### Preliminary results

Preliminary experiments were carried out to evaluate sample and solvent amount and eliminate solvents with lower efficiency. As the weight of *Ipomoea* seeds varies from 15 to 65 mg, a 10 mg sample was chosen, as it would enable analysis of a single seed. Then, using UAE-B, the following solvents were tested: methanol, acetonitrile, and methanol:water 1:1 mix with and without addition of 0.1 % formic acid. It was assessed that methanol and methanol:water mixture result in highest extraction efficiency. Therefore, during optimization of extraction methods, methanol:water mixture in different ratios was taken into consideration.

**Fig. 2:**
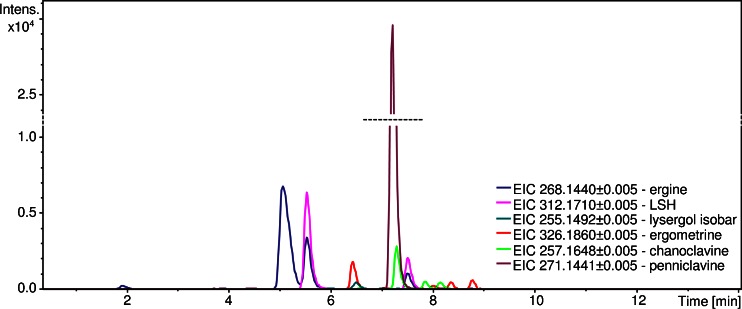
Analysis of the UAE-B extract of 10 mg of IT-HB1 seeds. Chromatograms acquired using the optimized LC-MS method. Ion chromatograms were extracted from the full scan spectrum

**Fig. 3:**
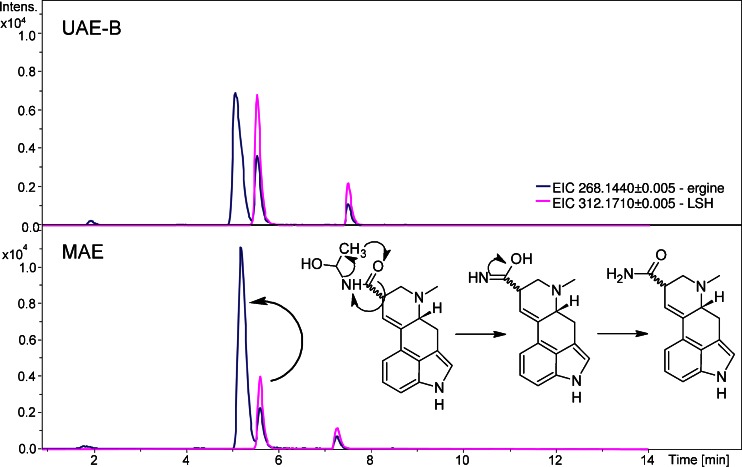
Effect of LSH decomposition after the MAE extraction of 10 mg of IT-HB1 seed sample, supported with hypothesized LSH to ergine reorganization mechanism. Ion chromatograms were extracted from the full scan spectrum

During preliminary experiments, six ergot alkaloids (Fig. 2): ergine, ergometrine, lysergic acid α-hydroxyethylamide (LSH), penniclavine, chanoclavine, and lysergol or one of its isobars (setoclavine or elymoclavine), together with their diastereoisomers, were identified in samples of IP-HB, IT-HB1, and IP-HB2. The results are shown in Table 1. Ergine and ergometrine were identified by comparing the retention time (RT) and parent ion *m/z*, and fragmentation spectra of standards and samples, and for the other compounds, identification was possible by comparing acquired fragmentation spectra to the detailed results already published [17, 18]. One should note that in the case of ergine, the first peak originates from ergine and isoergine and it has the same RT as ergine standard used in the experiments. Two following peaks have different RT than standard, but exactly the same as LSH. As those peak RTs overlap perfectly even if other chromatographic gradient programs or columns are used, it was hypothesized that LSH molecule rearranges into ergine due to too aggressive ionization conditions (Fig. 3) in a similar way that was described by Paulke et al. [17].

**Table 1:** Ergot alkaloids and their isoforms identified using LC-MS-Q-TOF

Compound	RT (min)	Calculated precursor *m/z*	Difference between calculated and experimental precursor^b^ Δppm	Fragment ions *m/z*
Ergine (LSA) and isoergine	5.06	268.144	0.9	208.081, 223.123, 237.104, 253.126
LSA isomer^a^	5.53			
LSA isomer^a^	7.50			
lysergic acid α-hydroxyethylamide (LSH) isomer 1	5.53	312.171	-0.3	208.080, 223.122, 237.098, 253.124, 268.144, 294.157
LSH isomer 2	7.50			
Ergometrine	6.42	326.189	2.8	208.077, 223.122, 251.118, 265.130, 283.143
iso-Ergometrine	8.00			
Ergometrine isomer 3	8.34			
Ergometrine isomer 4	8.76			
Lysergol or its isobars	6.49	255.149	-3.6	224.113, 240.123
Peniclavine	7.20	271.144	-7.2	222.091, 240.101, 253.132
Chanoclavine isomer 1	7.28	257.165	-3.6	208.111, 226.122
Chanoclavine isomer 2	7.84			
Chanoclavine isomer 3	8.13			

^a^Generated in the ion source

^b^Calculated using a SmartFormula program in ESI Compas 1.3 – Data Analysis 4.0 SP1 software (Bruker, Bremen, Germany)

Moreover, for 10 mg samples, 10 mL of solvent was sufficient to determine both ergine and ergometrine within linearity range of the LC-MS method. One should notice that none of the ergot alkaloids was found in samples of IP-MC and IP, even using 50 mg of seeds for extraction.

### Optimization of extraction method

The Doehlert experimental design with RSM was applied to optimize MAE and UAE-B techniques for maximum efficiency of ergine and ergometrine extraction.

In the case of microwave-assisted extraction, this approach seemed to work perfectly at first glance and enabled prediction of optimal extraction conditions. However, thorough analysis of results revealed that after MAE, the concentration of ergine in the extracts significantly increases, while the concentration of LSH decreases, especially compared with UAE-B and UAE-S. Moreover, the abundances of LSA and LSH are in inverse relationship. Indeed, as response models of both ergine and LSH concentration were calculated, it was assessed that the same factors were significant in both functions, but with inverse coefficients (data not shown). The reason for such phenomenon may be that microwave energy delivered to the system induces rearrangement of labile LSH to LSA, similarly to the process that is observed in ion source (see section “Preliminary results” and Fig. 3). This outcome indicated that MAE cannot therefore be used herein, as ergine should be accurately determined.

**Fig. 4:**
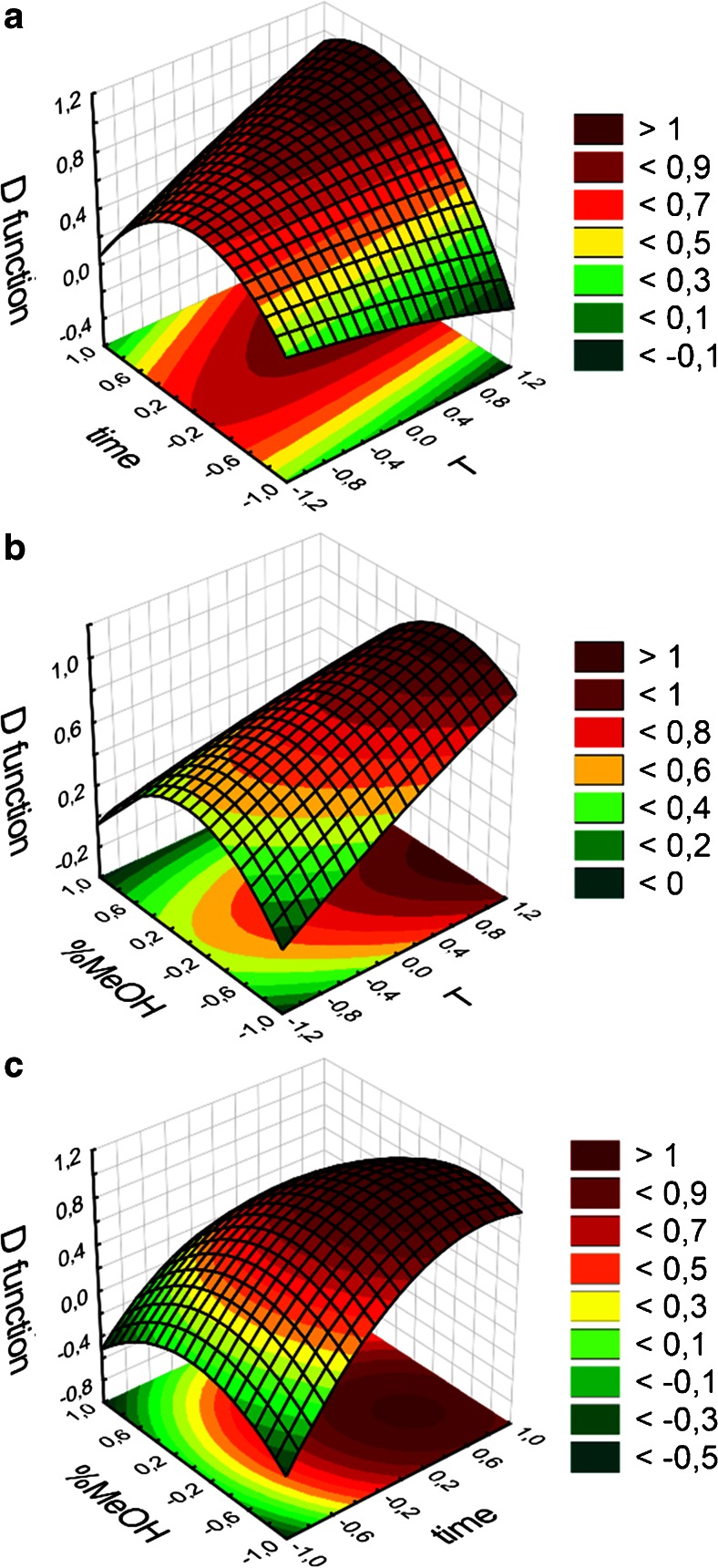
Response surfaces for UAE-B optimization using the Doehlert experimental design. D: desirability function; %MeOH: content of methanol in extracting solvent; T: programmed temperature of extraction; time: time of holding the programmed extraction temperature

Response surfaces for UAE-B optimization are presented in Fig. 4 as three 3-dimensional graphs of a 4-dimensional function. Please note the clear maximum of both the MeOH concentration and the time of extraction are visible on graph C. The maximum temperature of extraction, as depicted on graphs A and B, lies at the end of the examined temperature range (60 °C). Theoretically, higher extraction efficiency could be accessible for higher temperature; however, such experiment could not be conducted, as it is not feasible to use higher temperature of MeOH-water mixture in non-airtight vessels. Ultimately, optimized extraction parameters calculated from D function were: 60 °C, 30 min and methanol:water (7:3, v/v) extraction mixture. The extraction efficiency was evaluated by subjecting the same sample to the extraction procedure twice. After the second extraction, there were no peaks originating from ergot alkaloids visible on the extracted ion chromatogram, so it was assumed that extraction efficiency was close to 100 %.

Similarly, for UAE-S optimization, methanol:water (7:3, v/v) was used as extraction solvent and it was assessed that the best results, with extraction efficiency close to 100 %, are obtained after 1.5 min extraction. The temperature of solvent did not exceed 60 °C. Yet, because every sample had to be treated separately and there was no possible automation of the process, this technique was characterized by low throughput and, therefore, it was rejected for further development because of its unsuitability for analysis of a large number of samples.

### UAE-B/LC-MS method validation

Validation parameters for the UAE-B/LC-MS method are listed in Table 2. Repeatability and intermediate precision fits the range of acceptance within 15 % limit, whereas recovery and matrix effect fit within 80–115 % range for individual assays [26, 27]. The linearity of the method covers the range from 5.0 to 300 ng mL^–1^ for both ergine and ergometrine, which is an equivalent of 5.0 to 300 μg g^–1^ in relation to 10 mg sample. The LOD and LOQ values estimated using diluted standard solutions and spiked seeds free from analytes were consistent with each other. Particularly, concertation of 1.0 μg g^–1^ resulted in S/N > 3 (LOD) and 3.0 μg g^–1^ in S/N > 10 (LOQ), in case of both analytes. All of the validation parameters were considered sufficient for applying the developed method to analysis of *Ipomoea* seeds samples.

**Table 2:** Validation parameters of the UAE-B/LC-MS method

	Ergine				Ergometrine			
	Standard addition (μg g^-1^)				Standard addition (μg g^–1^)			
	+0	+80	+160	+240	+0	+10	+20	+30
Recovery (%)	-	91 ± 11	99 ± 12	106 ± 8	-	102 ± 8	104 ± 10	109 ± 3
Matrix effect (%)	-	105 ± 3	100 ± 5	94 ± 1	-	105 ± 4	94 ± 3	90 ± 3
Repeatability (%)	8.4	4.3	5.5	6.6	6.9	3.7	6.2	6.9
Intermediate precision (%)	11.8	11.8	9.4	11.0	1.1	5.0	5.5	1.1
Linearity (μg g^–1^)^a^; (ng mL^–1^)	5.0–300				3.0–300			
LOD (μg g^–1^) ^a^; (ng mL^–1^)	1.0				1.0			
LOQ (μg g^–1^)^a^; (ng mL^–1^)	3.0				3.0			

^a^Calculated with regard to 10 mg seed sample

### Analysis of different *Ipomoea* seeds

The UAE-B/LC-MS method was used for determination of ergot alkaloids in *Ipomoea* seeds (n = 3). For quantitative analysis of samples (10 mg each) from seeds packs, mean value with standard deviation of ergine or ergometrine concentration is given, whereas for single seed samples, concentration range is listed. Since there were no available standards for other identified ergot alkaloids, the relative abundance estimation was performed by calculating compound to IS areas ratio for the most abundant isomer of each compound to express differences between samples and Ipomoea cultivars. Results of quantitative analysis of *Ipomoea* seeds are listed in Table 3.

**Table 3:** Determination of ergine, ergometrine in Morning Glory seeds and relative abundance of other ergot alkaloids identified in the samples

Seeds	Sample	Concentration (μg g^–1^)^a^		Relative abundance - compound/IS area ratio^b^					
		Ergine	Ergometrine	Ergine RT = 3.37	LSH RT = 4.17	Lyzergol/isobars RT = 4.77	Ergometrine RT = 4.78	Penniclavine RT = 5.00	Chanoclavine RT = 5.05
IP-HB	Pack	300 ± 6	50 ± 2	2.45	1.09	0.11	0.41	4.21	0.43
	Single seed	<LOD - 537	<LOD - 93						
IT-HB1	Pack	261 ± 27	94 ± 8	2.13	0.54	0.14	0.77	4.75	0.42
	Single seed	3–502	<LOD - 109						
IT-HB2	Pack	297 ± 20	40 ± 3	2.43	1.71	0.16	0.33	5.08	0.85
	Single seed	255–495	34 – 93						

^a^ For quantitative analysis of samples from seeds packs, mean value with standard deviation is given, while for single seeds samples, concentration range is specified

^b^calculated for most abundant isomer

Although concentration of ergine in all three seed pack samples is similar and comparable to the one reported in the literature [6], it is clearly visible that in the case of single seeds, the amount of both ergine and ergometrine varies substantially, from concentration close or below LOD to two times greater than the mean concentration, which may create a risk of overdose for people experimenting with Morning Glory seeds.

Alkaloids abundance in all three HB cultivars is comparable, with most significant difference for LSH, which varies from 0.54 to 1.71 compound to IS ratio. As has been demonstrated in this study, LSH is a labile compound, and therefore the variances in its concentration may be due to different age and storage conditions of the seeds rather than difference in plant metabolism. Indeed, seeds IT-HB2, which express highest concentration of LSH, were bought directly from the producer, whereas seeds IP-HB1 were purchased in retail stores.

High relative abundance of penniclavine is an interesting observation; however, it cannot be directly connected with high concentration of this clavine. As there is significant difference in ionization efficiency of different compounds in ESI ion source, comparison with standard is required for assessing penniclavine concentration.

### Analysis of IP-HB2 young plants

The extracts of plant tissue (leaves with stems) were prepared in a similar manner to seeds extracts, with the difference of 50 mg of sample being used to provide higher alkaloids concentration in the extract. Extracts were purified with graphitized carbon by vortexing 1.5 mL of extract with 10 mg of carbon and subsequently centrifuged at 10,000 RPM for 5 min in room temperature, filtered through 0.45 μm regenerated cellulose syringe filter, diluted 1:1 with ultrapure water, and analyzed. The sample:carbon ratio was established on the basis of our unpublished work devoted to preparation of samples containing chlorophyll. It was also experimentally checked and confirmed that the graphitized carbon does not absorb ergine, ergometrine, or IS from the solution.

The found concentration of ergine and ergometrine were 24.6 ± 1.8 μg g^–1^ and 1.6 ± 0.1 μg g^–1^, respectively. Presence of investigated alkaloids in the tissue of glasshouse plants can be explained by probable fungal infection during cultivation. Endophytes can be transmitted through seeds (vertical path) or from the environment [31, 32], and the composition of species and their occurrence is dynamic and depends on many factors [33]. It is hard to track down how the fungi had colonized the examined plants. According to the presence of alkaloids in seeds (from the same source), the most likely is a vertical path, especially since ergine and ergometrine concentration is 12-fold lower in plant samples than in seeds. However, an old tropical greenhouse (est. 1954) with a big collection of plants is probably also rich in endophytic and epibiotic species of fungi.

## Conclusions

In this study, three extraction methods utilizing MAE, UAE-S, and UAE-B were assessed for identification and determination of ergot alkaloids in Morning Glory seeds. Only UAE-B has proven to be suitable for such analysis and the method was optimized in terms of temperature, time, and solvent composition. The developed UAE-B/LC-MS analysis method is fast, simple, requires only 10 mg of sample, and no additional extract treatment is needed. The use of ultrasound-assisted extraction enabled significant shortening of extraction time in comparison to similar seed samples [17]. The method was validated and proven to be suitable for the analysis of Morning Glory seeds from different sources.

It is worth mentioning that unexpected LSA concentration increase has been observed after the MAE extraction. However, it is known that microwave energy may induce the decay of analytes, which is one of the mayor drawbacks of the MAE technique discussed by Zhang et al. [34], but it rarely happens that the formation of target analyte is induced by microwaves. Once again it proves that the analyst needs to take a holistic approach on analytical process, as the unpredicted effects may disturb the validity of the results.

The analysis of seeds revealed that only three out of five kinds of seeds contained ergot alkaloids, all from “Heavenly Blue” cultivar, although from both *Ipomoea purpurea* and *Ipomoea tricolor* species (IP-HB, IT-HB1, and IT-HB2). Every time, ergine, ergometrine, LSH, penniclavine, chanoclavine, and their isomers were identified together with a compound that may be lysergol or one of its isobars. Concentration of ergometrine in samples representative for a given seed pack was comparable with results from literature, but concentration in single seeds varied substantially. A similar situation was observed for ergometrine concentration, but there is no data in the literature concerning mean concentration of this compound.

This phenomenon can be a serious concern in the case of people abusing *Ipomoea* seeds, as there is the possibility of overdosing by either accidentally selecting more potent seeds for ingestion or taking a second dose when the first was less effective than the average.

The developed method was successfully applied to the analysis of green parts of plants cultivated from IT-HB2 seeds. Ergine and ergometrine were both determined in the samples, but in lower concentration. The presence of alkaloids may be connected to endophytic fungi transmitted to the plant from its seeds or from the environment, although the first alternative is more probable.

## Electronic supplementary material

Below is the link to the electronic supplementary material.

ESM 1 (PDF 25 kb)
