# Supplementary material for: Identification and determination of ergot alkaloids in Morning Glory cultivars
Source: Anal Bioanal Chem. 2016 Feb 12;408:3093–102. doi: 10.1007/s00216-016-9322-5 (PMC4830885; doi:10.1007/s00216-016-9322-5)
Supplement: Supplementary file 1 — (PDF 25 kb) [file 216_2016_9322_MOESM1_ESM.pdf]

Analytical and Bioanalytical Chemistry

Electronic Supplementary Material

Identification and determination of ergot alkaloids in Morning Glory cultivars

Julia Nowak, Michał Woźniakiewicz, Piotr Klepacki, Anna Sowa, Paweł Kościelniak

Table S1 Doehlert experimental design for optimization of UAE-B. Coded and experimental parameters values

**Coded experiment values**				**Experimental parameters values**		
**No. of experiment**	**Factors**			**Factors**		
	**A**	**B**	**C**	**A T (°C)**	**B t (min)**	**C MeOH%**
1	0	0	0	50	20	75
2	1	0	0	60	20	75
3	0.5	0.866	0	55	35	75
4	0.5	0.289	0.817	55	25	100
5	-1	0	0	40	20	75
6	-0.5	-0.866	0	45	5	75
7	-0.5	-0.289	-0.817	45	10	50
8	0.5	-0.866	0	55	5	75
9	0.5	-0.289	0.817	55	10	100
10	-0.5	0.866	0	45	35	75
11	0	0.577	-0.817	50	30	50
12	-0.5	0.289	0.817	45	25	100
13	0	-0.577	0.817	50	15	100

Table S2 Doehlert experimental design for optimization of MAE. Coded and experimental parameters values

Coded experiment values				Experimental parameters values		
No. of experiment	Factors			Factors		
	A	B	C	A<br>T (°C)	B<br>t (min)	C<br>MeOH%
1	0	0	0	65	20	75
2	1	0	0	85	20	75
3	0.5	0.866	0	75	35	75
4	0.5	0.289	0.817	75	25	100
5	-1	0	0	45	20	75
6	-0.5	-0.866	0	55	5	75
7	-0.5	-0.289	-0.817	55	10	50
8	0.5	-0.866	0	75	5	75
9	0.5	-0.289	0.817	75	10	100
10	-0.5	0.866	0	55	35	75
11	0	0.577	-0.817	65	30	50
12	-0.5	0.289	0.817	55	25	100
13	0	-0.577	0.817	65	15	100
